# 2,6-Bis(2-chloro­benzyl­idene)cyclo­hexanone

**DOI:** 10.1107/S1600536809007648

**Published:** 2009-03-06

**Authors:** Deyun Liu

**Affiliations:** a Liaocheng Vocational and Technical College, Liaocheng, 252059, People’s Republic of China

## Abstract

In the title mol­ecule, C_20_H_16_Cl_2_O, the central cyclo­hexa­none ring adopts an envelope conformation. The two aromatic rings form a dihedral angle of 30.0 (1)°. The crystal packing exhibits weak inter­molecular C—H⋯O hydrogen bonds and short Cl⋯O contacts [3.213 (3) Å].

## Related literature

For general background, see: Tanaka & Toda (2000 ▶). For a similar crystal structure, see: Brinda *et al.* (2007 ▶).
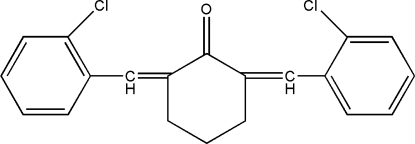


## Experimental

### 

#### Crystal data

C_20_H_16_Cl_2_O
*M* *_r_* = 343.23
Orthorhombic, 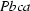

*a* = 14.4004 (15) Å
*b* = 8.1553 (10) Å
*c* = 28.593 (3) Å
*V* = 3358.0 (6) Å^3^
*Z* = 8
Mo *K*α radiation
μ = 0.39 mm^−1^
*T* = 298 K
0.42 × 0.32 × 0.17 mm

#### Data collection

Bruker SMART APEX CCD area-detector diffractometer
Absorption correction: multi-scan (*SADABS*; Sheldrick, 1996 ▶) *T*_min_ = 0.854, *T*_max_ = 0.937
13876 measured reflections
2962 independent reflections
1762 reflections with *I* > 2σ(*I*)
*R*_int_ = 0.064

#### Refinement

*R*[*F* ^2^ > 2σ(*F* ^2^)] = 0.045
*wR*(*F* ^2^) = 0.124
*S* = 1.06
2962 reflections
208 parameters
H-atom parameters constrained
Δρ_max_ = 0.24 e Å^−3^
Δρ_min_ = −0.32 e Å^−3^

### 

Data collection: *SMART* (Siemens, 1996 ▶); cell refinement: *SAINT* (Siemens, 1996 ▶); data reduction: *SAINT* (Siemens, 1996 ▶); program(s) used to solve structure: *SHELXS97* (Sheldrick, 2008 ▶); program(s) used to refine structure: *SHELXL97* (Sheldrick, 2008 ▶); molecular graphics: *SHELXTL* (Sheldrick, 2008 ▶); software used to prepare material for publication: *SHELXTL*.

## Supplementary Material

Crystal structure: contains datablocks I, global. DOI: 10.1107/S1600536809007648/cv2518sup1.cif

Structure factors: contains datablocks I. DOI: 10.1107/S1600536809007648/cv2518Isup2.hkl

Additional supplementary materials:  crystallographic information; 3D view; checkCIF report

## Figures and Tables

**Table 1:** Hydrogen-bond geometry (Å, °)

*D*—H⋯*A*	*D*—H	H⋯*A*	*D*⋯*A*	*D*—H⋯*A*
C20—H20⋯O1^i^	0.93	2.51	3.352 (4)	151

Symmetry code: (i) 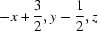.

**Fig. 1.**
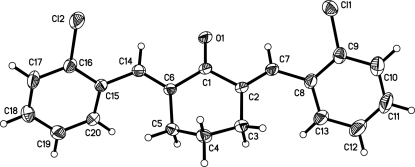
The molecular structure of (I) showing the atomic numbering scheme and 30% probability displacement ellipsoids.

## supplementary crystallographic information

### Comment

Development of new solid phase (solvent-free) reactions and transferring solution phase reactions to solid phase are subjects of recent interest in the context of generating libraries of molecules for the discovery of biologically active leads and also for the optimization of potent drug candidates (Tanaka & Toda, 2000).

In this paper, we describe the synthesis of the title compound, (I), starting from the fragrant aldehydes and cyclohexanone in the presence of NaOH under solvent-free conditions. This method can be considered as a general method for the synthesis of benzylidene cyclohexanones.

In (I) (Fig. 1), all bond lengths and angles are normal and correspond to those observed in 4-methyl-2,6-bis(2-naphthylmethylene) cyclohexan-1-one (Brinda *et al.*, 2007). The central cyclohexanone ring adopts an envelope conformation, the dihedral angles between the rings C8-C13 and C15-C20 is 30.0 (1)°.

The crystal packing exhibits short Cl···O contacts (Table 1) and weak intermolecular C—H···O hydrogen bonds (Table 2).

### Experimental

2-Chlorobenzaldehyde (2 mmol) and cyclohexanone (1.0 mmol), NaOH (2.0 mmol) were mixed in 50 ml flash under sovlent-free condtions After stirring 15 min at 293 K, tthe resulting mixture was washed with water for several times for removing NaOH, and recrystalized from ethanol, and afforded the title compound as a crystalline solid. Elemental analysis: calcd. for C_20_H_26_Cl_2_O: C 69.98, H 4.70%; found: C 69.93, H 4.65%.

### Refinement

All H atoms were positioned geometrically and refined using a riding model with C—H = 0.93–0.97 Å and *U*_iso_(H) = 1.2*U*_eq_(C).

### Crystal data

**Table:**

C_20_H_16_Cl_2_O	*D*_x_ = 1.358 Mg m^−^^3^
*M**_r_* = 343.23	Mo *K*α radiation, λ = 0.71073 Å
Orthorhombic, *P**b**c**a*	Cell parameters from 2653 reflections
*a* = 14.4004 (15) Å	θ = 2.8–43.8°
*b* = 8.1553 (10) Å	µ = 0.39 mm^−^^1^
*c* = 28.593 (3) Å	*T* = 298 K
*V* = 3358.0 (6) Å^3^	Needle, colourless
*Z* = 8	0.42 × 0.32 × 0.17 mm
*F*(000) = 1424	

### Data collection

**Table:**

Bruker SMART APEX CCD area-detector diffractometer	2962 independent reflections
Radiation source: fine-focus sealed tube	1762 reflections with *I* > 2σ(*I*)
graphite	*R*_int_ = 0.064
phi and ω scans	θ_max_ = 25.0°, θ_min_ = 1.4°
Absorption correction: multi-scan (*SADABS*; Sheldrick, 1996)	*h* = −14→17
*T*_min_ = 0.854, *T*_max_ = 0.937	*k* = −8→9
13876 measured reflections	*l* = −29→34

### Refinement

**Table:**

Refinement on *F*^2^	Primary atom site location: structure-invariant direct methods
Least-squares matrix: full	Secondary atom site location: difference Fourier map
*R*[*F*^2^ > 2σ(*F*^2^)] = 0.045	Hydrogen site location: inferred from neighbouring sites
*wR*(*F*^2^) = 0.124	H-atom parameters constrained
*S* = 1.06	*w* = 1/[σ^2^(*F*_o_^2^) + (0.0353*P*)^2^ + 3.2692*P*] where *P* = (*F*_o_^2^ + 2*F*_c_^2^)/3
2962 reflections	(Δ/σ)_max_ = 0.001
208 parameters	Δρ_max_ = 0.24 e Å^−^^3^
0 restraints	Δρ_min_ = −0.32 e Å^−^^3^

### Special details

**Table:**

Geometry. All e.s.d.'s (except the e.s.d. in the dihedral angle between two l.s. planes) are estimated using the full covariance matrix. The cell e.s.d.'s are taken into account individually in the estimation of e.s.d.'s in distances, angles and torsion angles; correlations between e.s.d.'s in cell parameters are only used when they are defined by crystal symmetry. An approximate (isotropic) treatment of cell e.s.d.'s is used for estimating e.s.d.'s involving l.s. planes.
Refinement. Refinement of *F*^2^ against ALL reflections. The weighted *R*-factor *wR* and goodness of fit *S* are based on *F*^2^, conventional *R*-factors *R* are based on *F*, with *F* set to zero for negative *F*^2^. The threshold expression of *F*^2^ > σ(*F*^2^) is used only for calculating *R*-factors(gt) *etc*. and is not relevant to the choice of reflections for refinement. *R*-factors based on *F*^2^ are statistically about twice as large as those based on *F*, and *R*- factors based on ALL data will be even larger.

### Fractional atomic coordinates and isotropic or equivalent isotropic displacement parameters (Å^2^)

**Table:**

	*x*	*y*	*z*	*U*_iso_*/*U*_eq_	
Cl1	1.01278 (6)	1.01596 (12)	0.34622 (3)	0.0568 (3)	
Cl2	0.72695 (8)	0.83897 (15)	0.07162 (3)	0.0783 (4)	
O1	0.86474 (15)	0.7337 (3)	0.22424 (7)	0.0515 (7)	
C1	0.7861 (2)	0.7354 (4)	0.24087 (10)	0.0342 (8)	
C2	0.7724 (2)	0.7802 (4)	0.29170 (10)	0.0347 (8)	
C3	0.6741 (2)	0.8022 (5)	0.30951 (11)	0.0472 (9)	
H3A	0.6726	0.8954	0.3306	0.057*	
H3B	0.6567	0.7058	0.3273	0.057*	
C4	0.6032 (2)	0.8289 (5)	0.27104 (11)	0.0455 (9)	
H4A	0.6145	0.9334	0.2559	0.055*	
H4B	0.5413	0.8313	0.2844	0.055*	
C5	0.6099 (2)	0.6916 (4)	0.23542 (11)	0.0399 (8)	
H5A	0.6006	0.5870	0.2509	0.048*	
H5B	0.5615	0.7046	0.2122	0.048*	
C6	0.7032 (2)	0.6928 (4)	0.21178 (10)	0.0335 (7)	
C7	0.8490 (2)	0.8002 (4)	0.31767 (10)	0.0412 (8)	
H7	0.9051	0.7861	0.3020	0.049*	
C8	0.8563 (2)	0.8412 (5)	0.36743 (11)	0.0458 (9)	
C9	0.9282 (2)	0.9410 (5)	0.38404 (11)	0.0485 (9)	
C10	0.9346 (3)	0.9856 (6)	0.43068 (13)	0.0654 (12)	
H10	0.9817	1.0555	0.4406	0.078*	
C11	0.8710 (3)	0.9260 (7)	0.46211 (14)	0.0806 (15)	
H11	0.8753	0.9550	0.4935	0.097*	
C12	0.8011 (3)	0.8238 (7)	0.44739 (14)	0.0836 (15)	
H12	0.7585	0.7832	0.4689	0.100*	
C13	0.7935 (3)	0.7809 (6)	0.40090 (12)	0.0626 (12)	
H13	0.7461	0.7108	0.3915	0.075*	
C14	0.7172 (2)	0.6681 (4)	0.16580 (11)	0.0399 (8)	
H14	0.7776	0.6812	0.1549	0.048*	
C15	0.6467 (2)	0.6227 (4)	0.13110 (10)	0.0382 (8)	
C16	0.6448 (2)	0.6917 (4)	0.08648 (11)	0.0444 (9)	
C17	0.5785 (3)	0.6500 (5)	0.05364 (12)	0.0561 (10)	
H17	0.5785	0.7002	0.0244	0.067*	
C18	0.5126 (3)	0.5339 (5)	0.06440 (13)	0.0610 (11)	
H18	0.4672	0.5062	0.0426	0.073*	
C19	0.5141 (3)	0.4588 (5)	0.10758 (13)	0.0588 (11)	
H19	0.4707	0.3781	0.1146	0.071*	
C20	0.5795 (2)	0.5029 (4)	0.14024 (11)	0.0461 (9)	
H20	0.5791	0.4515	0.1693	0.055*	

### Atomic displacement parameters (Å^2^)

**Table:**

	*U*^11^	*U*^22^	*U*^33^	*U*^12^	*U*^13^	*U*^23^
Cl1	0.0448 (5)	0.0640 (7)	0.0615 (6)	−0.0018 (5)	−0.0062 (5)	−0.0015 (5)
Cl2	0.1071 (9)	0.0832 (8)	0.0447 (5)	−0.0501 (7)	−0.0081 (6)	0.0118 (5)
O1	0.0311 (14)	0.086 (2)	0.0376 (13)	−0.0010 (13)	0.0065 (11)	0.0017 (12)
C1	0.0311 (19)	0.037 (2)	0.0346 (17)	0.0016 (15)	0.0042 (15)	0.0061 (14)
C2	0.0328 (19)	0.038 (2)	0.0338 (17)	−0.0009 (15)	0.0062 (14)	0.0039 (14)
C3	0.038 (2)	0.060 (3)	0.0431 (19)	−0.0041 (18)	0.0083 (16)	−0.0082 (17)
C4	0.0317 (19)	0.051 (2)	0.054 (2)	0.0072 (16)	0.0020 (16)	−0.0050 (18)
C5	0.0316 (19)	0.046 (2)	0.0419 (18)	−0.0017 (16)	0.0002 (15)	0.0013 (16)
C6	0.0296 (18)	0.037 (2)	0.0336 (17)	0.0031 (14)	0.0025 (14)	0.0066 (14)
C7	0.032 (2)	0.054 (2)	0.0376 (18)	0.0038 (16)	0.0037 (15)	0.0027 (16)
C8	0.045 (2)	0.058 (2)	0.0338 (18)	0.0056 (18)	−0.0014 (16)	−0.0029 (17)
C9	0.050 (2)	0.055 (3)	0.040 (2)	0.0114 (19)	−0.0070 (17)	−0.0024 (17)
C10	0.068 (3)	0.078 (3)	0.050 (2)	0.011 (2)	−0.011 (2)	−0.014 (2)
C11	0.085 (4)	0.120 (4)	0.036 (2)	0.017 (3)	−0.008 (2)	−0.015 (3)
C12	0.076 (3)	0.134 (5)	0.041 (2)	0.001 (3)	0.010 (2)	0.007 (3)
C13	0.058 (3)	0.091 (3)	0.039 (2)	−0.003 (2)	0.0029 (19)	0.006 (2)
C14	0.0346 (19)	0.045 (2)	0.0405 (19)	−0.0006 (16)	0.0013 (15)	0.0063 (16)
C15	0.0365 (19)	0.044 (2)	0.0337 (17)	0.0016 (16)	0.0025 (15)	−0.0040 (15)
C16	0.058 (2)	0.041 (2)	0.0345 (18)	−0.0080 (18)	0.0020 (16)	−0.0019 (15)
C17	0.077 (3)	0.060 (3)	0.0318 (18)	−0.007 (2)	−0.0099 (19)	−0.0037 (18)
C18	0.062 (3)	0.070 (3)	0.051 (2)	−0.014 (2)	−0.010 (2)	−0.015 (2)
C19	0.054 (2)	0.066 (3)	0.056 (2)	−0.018 (2)	0.004 (2)	−0.009 (2)
C20	0.049 (2)	0.053 (2)	0.0370 (18)	−0.0034 (19)	0.0034 (16)	0.0057 (17)

### Geometric parameters (Å, °)

**Table:**

Cl1—C9	1.739 (4)	C9—C10	1.385 (5)
Cl2—C16	1.738 (3)	C10—C11	1.372 (6)
O1—C1	1.228 (3)	C10—H10	0.9300
C1—C6	1.496 (4)	C11—C12	1.374 (6)
C1—C2	1.511 (4)	C11—H11	0.9300
C2—C7	1.340 (4)	C12—C13	1.379 (5)
C2—C3	1.514 (4)	C12—H12	0.9300
C3—C4	1.517 (4)	C13—H13	0.9300
C3—H3A	0.9700	C14—C15	1.467 (4)
C3—H3B	0.9700	C14—H14	0.9300
C4—C5	1.517 (4)	C15—C16	1.395 (4)
C4—H4A	0.9700	C15—C20	1.400 (4)
C4—H4B	0.9700	C16—C17	1.381 (5)
C5—C6	1.504 (4)	C17—C18	1.376 (5)
C5—H5A	0.9700	C17—H17	0.9300
C5—H5B	0.9700	C18—C19	1.378 (5)
C6—C14	1.345 (4)	C18—H18	0.9300
C7—C8	1.465 (4)	C19—C20	1.374 (5)
C7—H7	0.9300	C19—H19	0.9300
C8—C9	1.400 (5)	C20—H20	0.9300
C8—C13	1.405 (5)		
			
Cl1···O1^i^	3.213 (3)		
			
C9—Cl1—O1^i^	165.55 (13)	C10—C9—Cl1	117.4 (3)
O1—C1—C6	121.2 (3)	C8—C9—Cl1	120.8 (3)
O1—C1—C2	119.7 (3)	C11—C10—C9	119.6 (4)
C6—C1—C2	119.1 (3)	C11—C10—H10	120.2
C7—C2—C1	117.0 (3)	C9—C10—H10	120.2
C7—C2—C3	124.7 (3)	C10—C11—C12	120.2 (4)
C1—C2—C3	118.3 (3)	C10—C11—H11	119.9
C2—C3—C4	113.7 (3)	C12—C11—H11	119.9
C2—C3—H3A	108.8	C11—C12—C13	120.5 (4)
C4—C3—H3A	108.8	C11—C12—H12	119.8
C2—C3—H3B	108.8	C13—C12—H12	119.8
C4—C3—H3B	108.8	C12—C13—C8	121.1 (4)
H3A—C3—H3B	107.7	C12—C13—H13	119.4
C3—C4—C5	109.8 (3)	C8—C13—H13	119.4
C3—C4—H4A	109.7	C6—C14—C15	126.6 (3)
C5—C4—H4A	109.7	C6—C14—H14	116.7
C3—C4—H4B	109.7	C15—C14—H14	116.7
C5—C4—H4B	109.7	C16—C15—C20	116.0 (3)
H4A—C4—H4B	108.2	C16—C15—C14	122.0 (3)
C6—C5—C4	110.7 (3)	C20—C15—C14	121.9 (3)
C6—C5—H5A	109.5	C17—C16—C15	122.4 (3)
C4—C5—H5A	109.5	C17—C16—Cl2	118.3 (3)
C6—C5—H5B	109.5	C15—C16—Cl2	119.3 (3)
C4—C5—H5B	109.5	C18—C17—C16	119.6 (3)
H5A—C5—H5B	108.1	C18—C17—H17	120.2
C14—C6—C1	117.4 (3)	C16—C17—H17	120.2
C14—C6—C5	124.9 (3)	C17—C18—C19	119.7 (3)
C1—C6—C5	117.7 (3)	C17—C18—H18	120.2
C2—C7—C8	128.7 (3)	C19—C18—H18	120.2
C2—C7—H7	115.7	C20—C19—C18	120.2 (4)
C8—C7—H7	115.7	C20—C19—H19	119.9
C9—C8—C13	116.6 (3)	C18—C19—H19	119.9
C9—C8—C7	121.0 (3)	C19—C20—C15	122.0 (3)
C13—C8—C7	122.4 (3)	C19—C20—H20	119.0
C10—C9—C8	121.9 (4)	C15—C20—H20	119.0

Symmetry codes: (i) −*x*+2, *y*+1/2, −*z*+1/2.

### Hydrogen-bond geometry (Å, °)

**Table:**

*D*—H···*A*	*D*—H	H···*A*	*D*···*A*	*D*—H···*A*
C20—H20···O1^ii^	0.93	2.51	3.352 (4)	151

Symmetry codes: (ii) −*x*+3/2, *y*−1/2, *z*.
